# Blockchain Based Delay and Energy Harvest Aware Healthcare Monitoring System in WBAN Environment

**DOI:** 10.3390/s22155763

**Published:** 2022-08-02

**Authors:** Helen Sharmila Anbarasan, Jaisankar Natarajan

**Affiliations:** School of Computer Science and Engineering, Vellore Institute of Technology (VIT), Vellore 632014, India; helen.sharmila2016@vitstudent.ac.in

**Keywords:** wireless body area networks, internet of things, blockchain, secure cluster based routing, quality of service (*QoS*)

## Abstract

Wireless body area networks (WBANs) are a research area that supports patients with healthcare monitoring. In WBAN, the Internet of Things (IoT) is connected with WBAN for a smart/remote healthcare monitoring system in which various medical diseases are diagnosed. Quality of service (*QoS*), security and energy efficiency achievements are the major issues in the WBAN-IoT environment. Existing schemes for these three issues fail to achieve them since nodes are resource constrained and hence delay and the energy consumption is minimized. In this paper, a blockchain-assisted delay and energy aware healthcare monitoring (B-DEAH) system is presented in the WBAN-IoT environment. Both body sensors and environment sensors are deployed with dual sinks for emergency and periodical packet transmission. Various processes are involved in this paper, and each process is described as follows: Key registration for patients using an extended version of the PRESENT algorithm is proposed. Cluster formation and cluster head selection are implemented using spotted hyena optimizer. Then, cluster-based routing is established using the MOORA algorithm. For data transmission, the patient block agent (PBA) is deployed and authenticated using the four Q curve asymmetric algorithm. In PBA, three entities are used: classifier and queue manager, channel selector and security manager. Each entity is run by a special function, as packets are classified using two stream deep reinforcement learning (TS-DRL) into three classes: emergency, non-emergency and faulty data. Individual packets are put into a separate queue, which is called emergency, periodical and faulty. Each queue is handled using Reyni entropy. Periodical packets are forwarded by a separate channel without any interference using a multi objective based channel selection algorithm. Then, all packets are encrypted and forwarded to the sink nodes. Simulation is conducted using the OMNeT++ network simulator, in which diverse parameters are evaluated and compared with several existing works in terms of network throughput for periodic (41.75 Kbps) and emergency packets (42.5 Kbps); end-to-end delay for periodic (0.036 s) and emergency packets (0.028 s); packet loss rate (1.1%); residual energy in terms of simulation rounds based on periodic (0.039 J) and emergency packets (0.044 J) and in terms of simulation time based on periodic (8.35 J) and emergency packets (8.53 J); success rate for periodic (87.83%) and emergency packets (87.5%); authentication time (3.25 s); and reliability (87.83%).

## 1. Introduction

A wireless body area network (WBAN) is a kind of sensor network that assists in resourceful patient healthcare monitoring. WBANs are also referred to as body sensor networks (BSNs). Many studies have recently been conducted in WBAN for continuous patient monitoring [1,2,3,4,5]. Unfortunately, the current WBAN does not meet the *QoS* for a patient [6,7]. *QoS* provisioning is an important consideration in WBANs that is achieved by resolving three challenges: energy efficiency, mobility prediction (adaptiveness) and security [8,9,10]. In general, WBAN communications are classified into three layers of communication: (1) intra-WBAN, (2) inter-WBAN and (3) beyond WBAN. Recent studies discussed any one of the WBAN communications [11].

Integration between WBAN and IoT provides remote health monitoring services for patients [12]. Due to the limited resources of IoT sensors, they must be efficiently utilized, but solving this issue is a critical issue. In particular, when moving the patient’s body, connectivity among sensor nodes and the sink node fails. This causes massive packet losses, and critical packets are dropped [13]. Clustering is one of the solutions to meet the energy consumption issue. In this approach, either a sink node or the best sensor node in the human body can act as a cluster head (CH). Through the CH, medical data packets are forwarded. This approach requires optimum and fast CH election to aggregate and transmit the packets [14]. Similar to energy efficiency, security is the biggest challenge in WBAN because nodes are communicated via a public channel, so it is vulnerable [15,16,17]. Blockchain is a promising solution to provide adequate security for WBAN [18,19,20]. It acts as a decentralized entity to avoid a single point of failure. However, the traditional blockchain architecture fails to provide data confidentiality for the resource-constrained environment.

Blockchain technology is referred to as the transparent distributed technology in the networking domain in which all the participants can own a ledger. Blockchain technology is also referred to as a peer-to-peer method in which all the participants collaboratively manage the network. Blockchain technology has provided the following advantages such as interoperability, scalability, data integrity and traceability [21,22]. There are four types of blockchain:

- Public blockchain: All participants can have rights to read and write, no centralized entity, high credibility and also high throughput and energy consumption.
- Private blockchain: Only the particular organization that owns the blockchain could have rights to access or write. It is a centralized blockchain with less credibility. It consumes less energy with a high throughput.
- Consortium blockchain: It can be owned by a group of organizations to which all the group organization participants could have access to read and write. It is also referred to as a partially centralized blockchain and has less energy consumption with a high throughput.
- Hybrid blockchain: Similar to the public blockchain, a hybrid blockchain could give access to read and write to all the participants. It provides partial centrality with medium credibility.

Due to the support of blockchain-based secure systems, medical records cannot be tampered with and protected from unauthorized access [23,24,25,26]. The proposed work also adopts blockchain technology for secure data storing and managing purposes because the energy efficiency may be reduced by the security threats.

### 1.1. Motivation and Objectives

The major aim of this research is to perform energy efficient health monitoring with low delay and high security. In WBAN, a patient’s remote healthcare monitoring system design invoked various challenges [27]. Current WBAN approaches do not ensure end-to-end security, low energy consumption and can cause delays because it is achieved either with intra-WBAN or beyond WBAN. When WBANs communicate with the same channel simultaneously, then they interfere with each other. For critical data transmission, an idle channel is required and periodical data requires the best channel to transmit it without any interference. Due to single-hop communication for the critical data, high energy consumption and end-to-end delay occur. At the single hop, the contention window size (*CWS*) must be adjusted to transmit packets according to *QoS* constraints. None of them considered environment-related data on a patient to monitor their health. We are motivated by the following problems that occur during health monitoring in which the major issues are listed as follows,

- Higher energy consumption: Frequent sensor replacement is necessary when sensors drain their energy.
- Security and privacy: Due to wireless channels and limited energy resources of sensors, data confidentiality and privacy are a failure.
- Mobility prediction: Communications must be energy-efficient and secure in patient dynamic movements.

This research objectives designed in this paper are as follows,

- To propose an energy-efficient, secure and delay-aware health monitoring system that allows patients to sense and transmit data in an energy-efficient manner.
- To ensure *QoS* constraints while collecting and transmitting the data from all layers (intra-WBAN, inter-WBAN and beyond WBAN).
- To safely store the sensed data in a storage server with satisfying the security requirements (data confidentiality, and integrity).

In this paper, we proposed a blockchain-assisted delay and energy-aware healthcare monitoring system for the early transmission of emergency packets to the respective authority to achieve these objectives.

### 1.2. Research Contributions

This work mainly focuses on energy efficient health monitoring with low delay and high security. The major contributions of this paper are sorted as follows,

- We deployed dual sinks such as emergency sink and periodic sink to collect the data without congestion. All critical packets are forwarded to the health center without any delay.
- All patients who need to monitor healthcare are registered to the key management server (*KMS*) using the extended PRESENT algorithm. Clustering is generated using the best objective function (BOF), which is computed by node residual energy, transmission power, bandwidth, and signal to noise ratio (SNR) via spotted hyena optimizer (SHO).
- Faulty data packets are removed in the sink with the use of analyzing the records and it results in low bandwidth and energy consumption. If the CH is drained of its potential to transmit the data, then the next node is selected among the patient body sensors.
- The idle channel is used for emergency packet transmission and selects the optimum channel for the remaining two medical packet types by considering RSS, SNR, channel capacity and radio power. Hence, interference is eliminated in this step.
- We transfer each normal data packet via multihop transmission, which considers packet size, data traffic type, *TTL*, required delay and data rate. A multi-hop data routing using best forwarders selection by considering node residual energy, RSS, path duration and distance to the CH.
- We sense and classify the environment and healthcare data for each patient using the deep reinforcement learning algorithm. We proposed two stream-DNN in DRL, which outperforms the conventional Markov model.
- We propose a PRESENT algorithm for encryption of medical data and critical data directly transmitted to the CH and then forwarded to the PBA. Finally, data packets are stored in the blockchain and cloud servers based on packet sensing type.

### 1.3. Paper Layout

The rest of this paper is organized as follows: Section 2 describes the existing research works and their deficiencies. Section 3 describes the main issues determined and emerging in this environment. Section 4 details all the defined solutions that solve security, *QoS* and energy efficiency issues. Section 5 demonstrates the experimental evaluation of the proposed work in comparison to the existing works, and Section 6 concludes the proposed solution with the extension of potential future research directions.

## 2. Review of Related Literature

This section illustrates the previous works studied for secure data transmission for patient healthcare monitoring in a WBAN-based IoT environment. Most of the works have concentrated on cryptography-based security mechanisms and less focused on obtaining the minimum energy consumption for body and environment based sensors.

### 2.1. QoS Achievement in WBAN/Iot

The authors in [28] present a WBAN architecture with dual sink nodes. Line of sight (LoS) and non-line of sight (NLoS) based clusters were formed, and then two sinks were placed. The major objective of this paper was to minimize the energy consumption of the network. After the data are sensed, critical data are sent to the sink directly, and periodical data are sent through the next forwarders. Equal time slots are assigned for all sensors deployed on a patient, which must be slotting time according to the node priority. An IoT-based BAN network architecture is presented to monitor human health [29]. Initially, sensed data were classified and assigned a priority level. Based on the priority level, data are routed to the sink node. The THE (temperature heterogeneity energy) protocol is tested for a WBAN with eight different sensors, including a temperature sensor, insulin pump, SPO2 sensor, EMG sensor, CGM sensor, BP sensor, EEG sensor and ECG sensor. To transmit periodical data, two hops are used as the relay nodes, but an optimal selection of relay nodes is important. In [30], the authors aimed to achieve energy efficiency, reliability and transmission efficiency. To achieve these aims, a multiobjective based routing protocol is designed by considering residual energy, bandwidth, transmission rate and hop count to the sink node. Routing is executed based on the maximum benefit function. This weight function is adjusted according to data priority. In this paper, the next forwarder is dynamically selected for data transmission. Data classification is significant to perform before data routing because successful data transmission shows high *QoS* performance. A priority-based MAC protocol is designed [31] to show better performance achievement for WBAN. A prior knowledge-based weighted routing algorithm is presented to choose the optimum route, and it is computed by residual energy, distance, delay and link stability. To further reduce energy consumption, graph-based sleep scheduling is proposed. In the coordinator node, a split and map-based NN is elaborated to perform packet classification. Then, packets are sent to the respective sink node based on the packet size, data traffic type and *TTL*. To forward data packets to the corresponding sink node, *QoS* constraints such as delay, bandwidth and data rate are needed. A queuing game approach is proposed to transmit delay-sensitive medical data beyond WBAN [32]. The proposed approach addresses the transmission of random arrival of medical packets on each gateway. At each gateway, packets are classified into multiple classes: one class specifies the emergency alarm, and other classes specify the nonmedical classes. Due to the consideration of several *QoS* constraints, packets are queued according to their classes. At each smart gateway, running the game theory approach consumes more time and causes more waiting time to send the critical data packets. Network modeling with BSs and multiple gateways leads to high energy consumption and packet loss ratios.

The authors in [33] focus on beyond WBAN communications to handle the massive medical data packets from each WBAN. A queuing system is designed to classify large medical data packets into multiple classes. It is based on the time and priority of the data packets. To address a relaxed queuing problem, an optimal scheduling scheme is presented. The drawbacks in this work are:

- It is complex to design a large scale WBAN environment since medical packets are gathered at each gateway in a random way, which leads to large end-to-end delay.
- Some of the medical packets are necessary to meet the *QoS* requirements, especially delay, which must be met by the predefined period of time.

In [34], two significant parameters are considered for the best next hop selection to route medical data packets to the BS. The link quality and energy utilization rate are computed for each on-body sensor. A sensor with high link quality and residual energy is selected as the next hop. The drawbacks in this work are limited metrics to select the next hop and not suited to transmit critical data.

An optimized health monitoring framework was introduced in [35]. This work consists of entities such as cluster members, cluster heads and core networks. In this work, the cluster head is selected by introducing three optimization algorithms, namely, ant colony optimization, multi objective particle swarm optimization and the comprehensive learning particle algorithm. The selected cluster head acts as a gateway between the core network and members in the clusters. This work employs a cluster head as a gateway between the core network and members in the clusters, which affects the *QoS* constraints in terms of latency and throughput.

### 2.2. Security in WBAN/Iot

A secure healthcare monitoring scheme is employed [36] to monitor and control the smart healthcare system. To design a secure scheme, the authors integrated two artificial intelligence algorithms, a fuzzy system and neural network classifiers. It decides the priority level of the data based on the collected sensor’s information. From the patient body, collected sensor data are sent via the GSM module to the Azure IoT hub. Secure communications have not been achieved successfully, which increases the security loss of the network. A secure and efficient anonymous authentication scheme was presented in [37] to preserve the location privacy of WBAN patients. The author proposes the Chinese remainder theorem (CRT) to keep the user’s location details more secret from unauthorized attackers. In this scheme, both doctors and patients are authenticated with their location to protect it. Experiments proved that the proposed work outperformed the impersonation attack, message modification attack, replay attack, man-in-the-middle attack and eavesdropping attack. To keep the location private for both the doctor and patient, the TA must be decentralized because when the TA fails to provide security, then the whole security is lost. A secure certificateless biometric authentication with group key management is presented in [38] for WBAN. However, the coordinator node plays a vital role in collecting the medical data because it acts as the personal controller for each WBAN. For authentication, electrocardiogram (ECG) records are used as the biometric feature. Then, group key management is executed for all validated sensor nodes in the WBAN. Lack of security and privacy due to ECC and one-way hash function is not suited for resource constrained IoT sensors.

A two level lightweight method is presented [39] for determining anomaly data from massive sensor readings. First, the game theory approach is applied to find the spatial correlation between the sensor readings and determine the dynamic changes in the WBAN. Second, the Mahalanobis distance is presented in the local processing unit (LPU), which has a global view in the multivariate analysis. The overall trust for sensors is very poor, and there is higher energy consumption in fault detection.

The authors in [40] introduced a secure framework for WBAN-IoT in healthcare systems. This work employs five layers, namely, the collection of the data layer from the WBAN sensors to the gateway agent, the second layer is responsible for data routing to the local gateway server from the gateway agent, the third layer is responsible for the routing of data to the distant server, and the fourth layer is responsible for data routing to the fog layer where controllers are deployed for providing security to the data and classify the data as critical and noncritical. Finally, all the data are stored in the private cloud. This work provides security to the data in the fog layer; however, the authenticity of the users was not considered, which leads to security threats and various attacks in the networks.

### 2.3. Blockchain in WBAN/Iot

The BSN energy efficient and secure scheme is presented in [41] heterogeneous sensor networks. All biosensors in the network use a certificateless cryptography scheme to solve security and privacy issues. A signcryption (online or offline) method is considered to reduce the difficulty in sensors. If patient data are known, then an online process is implemented, whereas the offline process is executed if the prior knowledge is unavailable. For secure patient transactions, hybrid blockchain technology is involved. More energy consumption and high computations are required while processing in the online phase.

A new storage model was studied in [42] to store the collected data using blockchain technology in a WBAN. In the blockchain, a designated verifier-based sequential aggregate signature scheme is presented to guarantee that the medical records can be viewed by the corresponding WBAN patient, i.e., privacy is protected to unauthorized patients. The main concern of this paper is to reduce the energy consumption for data computations and transmission. Likewise, the main novelty of this paper is to minimize the storage space of the block by generating the signature. Data storage in blockchain improves the storage space, but security is not stronger. Blockchain technology is focused on the eHealthcare management system to provide the interoperable WBAN [43]. This system follows the IEEE 802.15.6 specification, and the proposed system has provided the advantages of a high security protection level, low hardware utilization, and stable performance. This is well suited for a large-scale environment, but emergency packets are delayed more. A secure cloud server-based health data auditing [44] is implemented in blockchain technology. For this purpose, a third-party auditor (TPA) is created to play the role of public auditing in which the integrity of the outsourced medical packets is verified periodically. In this paper, a decentralized security framework is added, which is integrated into the data auditing element for security provisioning and is referred to as the Ethereum blockchain technique. In addition to TPA, the private key generator is deployed to generate the public and private keys. Each health record is maintained in TPA and is transferred into Ethereum. It is not suitable for resource-limited WBANs, and its performance is high when small sensors are deployed.

Table 1 illustrates the overall research gaps in the WBAN-IoT that are identified from the in-depth literature in terms of *QoS*, energy wastage, and security, which are solved by the proposed solution, and the gaps are filled with the best solutions for *QoS* achievement in WBAN-IoT.

## 3. Problem Statement

This section summarizes the main problems that exist in the current literature on WBAN-based IoT. In [45], the authors objective is to minimize the energy consumption for all sensor nodes deployed in the human body. To meet this objective, the energy harvested aware routing protocol (E-HARP) was proposed. In the E-HARP, two tasks are executed such as dynamic CH election and data routing in a cooperative manner. Initially, CHs are elected by the cost factor (CF), which is calculated by the node residual energy, transmission power needed, signal-to-noise ratio (SNR) and energy loss. The problems in this work are as follows,

- The clustering process is not effective since it does not ensure that the CF computation always produces the optimum solution and the computation of CF tends to be time consuming when a large number of sensors are used. This paper minimizes the redundant data, but it does not eliminate anomaly (faulty) data. Thus, it results in large bandwidth and energy consumption for CHs.
- When two sensors communicate with the same channel concurrently, they interfere with each other. Multihop transmission via nonoptimal nodes increases energy consumption and reduces the delivery rate.

Then, the optimal cost function (CF) was computed [46] in the E-HARP. The optimal cost function was computed using thelLink SNR, transmission power required, distance between nodes and total energy harvested and residual. Two sink nodes are deployed according to the LOS and high *RSSI* value, and these nodes act as CHs. However, a patient’s critical packets tend to be waiting for a longer time, and thus energy consumption is higher. It is not suitable for large scale environments. It is required to sense the environment because of the large size of faulty packets generated in body sensors, which also consume more energy.

A remote monitoring system was presented in [47], which contains a patient centric agent (PCA) that collects and processes the sensed packets from body sensors. The PCA component is an entity deployed by the blockchain and preserves data privacy. When the distance of the critical sensors is far from the SDP, energy consumption is very large because body sensors directly send the sensed data to the SDP and then to the PDA. It is not appropriate to transmit emergency data packets since the use of SDP intermediately causes a longer delay when delivering critical data to the PCA. These packets are encrypted using the AES counter mode (AES-CTR). In this algorithm, the bit-flip error is high, and after decryption, it is presented in some of the blocks of ciphertext. If a counter value is inappropriately used, then all security is lost. Delay aware scheduling is executed for various kinds [48] of medical packet transmissions in IoT-based healthcare applications. This paper focuses on beyond WBAN communication, which is data transmissions between gateways and the base station to the remote monitoring system. For scheduling, medical packets are sorted according to the priority awareness and delay constraints. However, a single BS collects all the sensed packets from K-gateways, which is ineffectual to collect all packets. A single BS suffers from large network density, handling different traffic flows and higher energy consumption.

- Each medical packet is transferred to the BS based on delay constraints. For example, the glucose monitoring delay requirement is <20 ms. When it is transmitted frequently, then the other delay-constrained emergency packets are dropped. If a medical packet has waited more than the delay time, then the packet is dropped from the scheduler.
- The Markov chain does not perform well when the actions are independent and it does not learn the environment to find the current state. Especially, it is needed for WBAN.
- The participation of multiple sensor nodes (256 heterogeneous sensors) causes congestion in data transmission and thus fails to transmit critical data or is dropped.

A robust authentication scheme was presented in [49] for patient authentication. User smart cards, biometric features and pseudo identities are used, which are held in gateway for each patient. In this step, the timestamp was used to verify the user authentication. This paper uses one-way (1 W) hashing for user credential hashing. In this work, the user authenticated to GW via a public channel, and thus, it is vulnerable to obtaining patient’s medical data. To compute the new password, a smart card is necessary. If the smartcard is lost, then it is possible to change the password by any hackers. A one-way hash does not provide high security since the hashed data can be retrieved easily.

## 4. System Model

In this research, we address the energy efficiency and security issues for *QoS* provisioning in the IoT-WBAN environment. Hence, we designed Energy and Delay aware medical data transmission framework in an IoT based WBAN.

### 4.1. System Model

**Figure 1:**
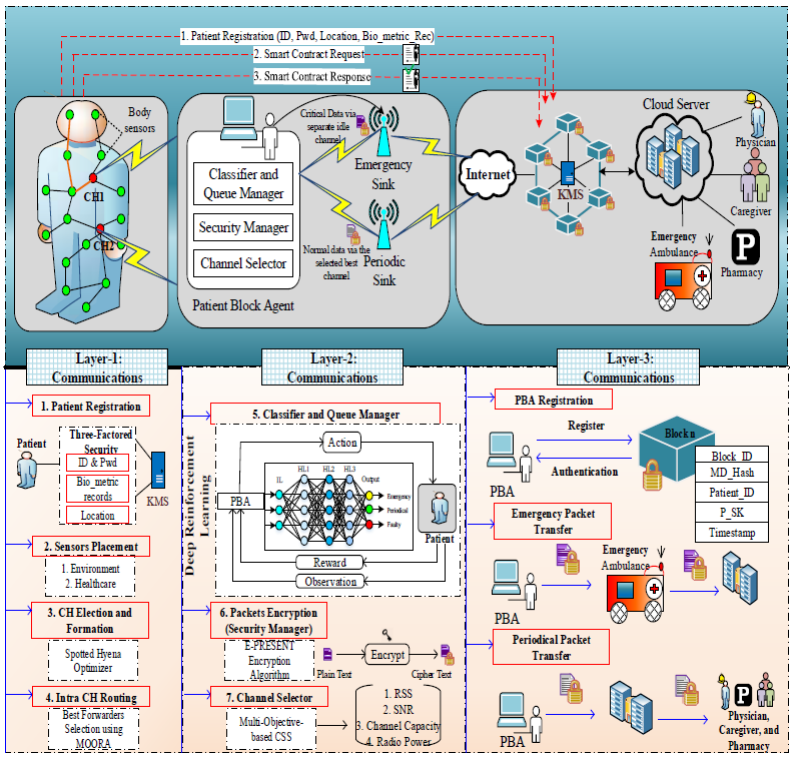
System architecture.

The overall system architecture is depicted in Figure 1. Three layers are designed in this work, including layer 1: Intra-WBAN communications, layer 2: inter-WBAN communications, and layer 3: beyond WBAN. Our proposed work consists of entities such as:

- *Body sensors*: The body sensors $\left(B_{s}=B_{s1},B_{s2},\cdots B_{sn}\right)$ are deployed in patients’ bodies to monitor the patient health conditions which monitor such as heartbeat rate, BP rate, etc.
- *Environmental sensors*: The environmental sensors $\left(E_{s}=E_{s1},E_{s2},\cdots E_{sn}\right)$ are located in the environment to monitor the environmental changes based on the patient’s health condition. These sensors monitor the air quality, humidity, etc., in the surroundings of patients’ environment.
- *Patient block agent (PBA)*: The patient block agent is responsible for the transmission of data and also provides authenticity to the users in which three modules are involved namely classifier and queue manager, security manager, and channel selector for classifying and securing the data.
- *Emergency sink and periodic sink*: These sinks are responsible for storing the data on the internet and cloud server. The emergency sink $\left(S_{E}\right)$ is used to share the critical data from the PBA while the periodic sink $\left(S_{P}\right)$ is used to share the normal data from the PBA which helps to transmit the health information with low delay and congestion.
- *Blockchain(key management server—KMS)*: Blockchain is used for the security and privacy of the WBAN IoT networks. The *KMS* in the blockchain is responsible for managing the key from the users during registration and authentication to verify the legitimacy of the users.

### 4.2. Key Distribution

We assume that patients move from one place to another dynamically. For this case, we design a secure, energy-efficient and delay-aware healthcare monitoring system for WBANs. We registered each patient to the *KMS* by their *ID*, *Pwd*, location and biometric records. Patient $P_{i}$ needs to submit {ID,PW} to the *KMS*. The *KMS* first verifies ID and Pwd for the corresponding patient. When ID and Pwd are correct, then the *KMS* verifies the patient’s current location and biometric records. Finally, SK is generated for registered patients. This step is formulated as follows,

(1) $$U_{i}\to \left\{ID⊕PW\right\}\to KMS$$

(2) KMS→{SK(P),if(ID&&PW&&BR&&L==True)}

**Figure 2:**
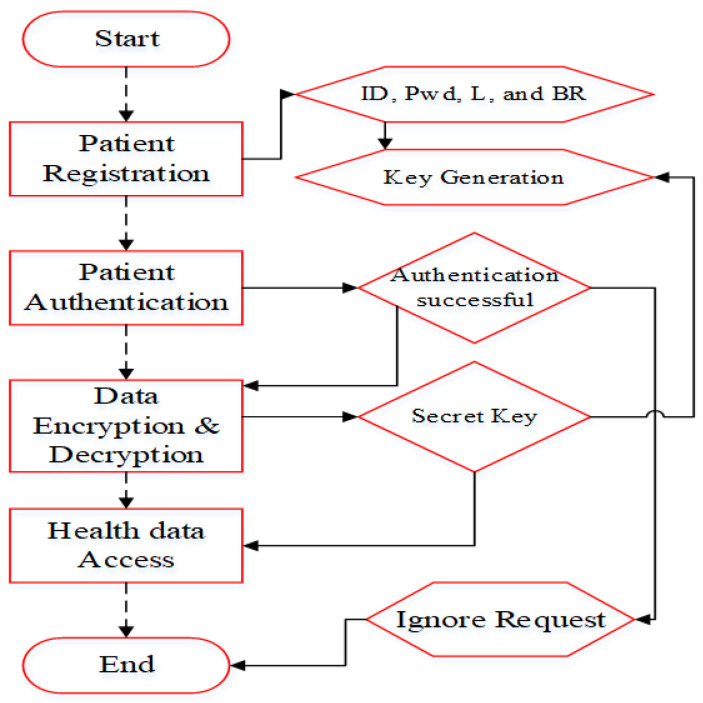
Security evaluation for WBAN.

If the patient is successfully registered, the secret key (*SK*) is generated and forwarded to them and is also stored in the PBA. For authentication, we verify these entities. For authorized patients, data transmission is implemented, and data access is allowed for patients who have the secret key. A flow of security provisioning is represented in Figure 2. The encryption and decryption process is explained in the following sections.

### 4.3. Cluster Based Routing

We form clusters from the sensors deployed in the patient. Table 2 describes the number of IoT devices used for patient monitoring.

Cluster head is implemented by computing the best objective function (BOF), which works out by node residual energy, transmission power, bandwidth and SNR via spotted hyena optimizer (SHO) [50]. In the MAC protocol, the data traffic type field is added, which shows the five different packets. When the sensor senses an emergency packet, then it will be directly sent to the CH. For the other four different packets, the optimum route is selected. We propose multihop data routing using optimum forwarder selection by considering the node residual energy, RSS, path duration and distance to the CH. The SHO algorithm finds the best CHs using four stages: searching, encircling, hunting and attacking prey. Based on the proper balancing between exploration and exploitation, the optimum CHs are determined. When the spotted hyena’s cooperation is higher, then the fitness values among sensors are also high.

The fitness is computed for the following parameters,

- **Residual Energy Level**: This parameter denotes the remaining amount of energy of the node. It is computed using the difference between the initial energy level and the total energy consumed after one round. A node with a higher residual energy level becomes CH and hence the CH is responsible for data collection and aggregation.
- **Distance**: This parameter is well-known in that it means the distance between one body sensor and the nearby sensor. A node with less distance is applicable for CH election. It is calculated as follows:
  (3) $$D_{s}=\sqrt{{\left(X_{i}-X_{j}\right)}^{2}+{\left(Y_{i}-Y_{j}\right)}^{2}}$$
- **Path Duration**: This parameter is the amount of time taken for packet transmission. It is measured using a number of relay nodes to one node.

The computation of RSS is explained in the following section. A node with higher residual energy, less distance, less path duration and high RSS is selected as the CH. The remaining nodes are connected based on their values. To select the route, we applied multi-objective optimization on the basis of ratio analysis (MOORA). It is a decision making algorithm that selects one or more alternatives from a set of available nodes. The first stage of the MOORA algorithm is to construct the decision matrix DM for the given problem. The objectives and alternatives are listed in the M×N matrix and the performance of *DM* is dependent upon the alternatives and also on the input parameters. It is expressed as follows,

(4) $$X=DM{\left(i,j\right)}_{M\times N}=\left[\begin{array}{ccc} x_{11} & x_{12} & x_{1n}\\ x_{21} & x_{22} & x_{2n}\\ x_{m1} & x_{m2} & x_{mn} \end{array}\right]$$

$x_{ij}$ is the performance value of the *i*th alternative on the *j*th criterion, and *M* and *N* are the alternative rows and columns, respectively. Then, the alternative performance value is computed against the other criteria that can be calculated as follows:

(5) $$x_{ij}^{*}=\frac{P_{ij}}{\sqrt{\sum_{k=1}^{m}P_{kj}^{2}}}$$

where $x_{ij}^{*}$ is a range between 0 and 1 and it produces the normalized performance for each input criterion.

The normalized values for the criteria are computed using nonbeneficial criteria. The sum of the nonbeneficial criteria is subtracted from the beneficial criteria. The result is the overall score value for the alternative. Attaining the best set of alternatives is the termination process of the MOORA algorithm. The special feature of the MOORA algorithm is to determine the best alternative by multiple objectives. Alternative values are arranged in ascending order, and the best node is selected based on the alternative value. In previous works, the sensor node sends sensed information through single hop communication. However, this type of communication is not available at all times, whereas some existing works have used multihop communication in which next forwarder selection was not optimal. Hence, packet losses are very high. Furthermore, this can be applied when a large number of sensors are placed over the body. Our proposed routing protocol computes the weight value for the best forwarder selection.

### 4.4. Contention Window Size Adjustment

In addition, we adjust the contention window size (*CWS*) by *QoS* factors: distance, *RSSI* and residual energy. This process reduces the end-to-end delay and energy consumption for transmitting emergency packets. Table 3 shows the data traffic specification. All the data are prioritized based on health parameters such as temperature, heart rate, blood pressure, respiration, ECG, etc. Based on these parameters, the data traffic type is classified into three types, as mentioned in Table 3. Depending upon the values of the health parameters, the data are queued to any one of the three queues.

### 4.5. Patient Block Agent

PBA plays a vital role in this research. It acts as a coordinator that is authenticated to the blockchain using the four-Q-curve algorithm. It is an asymmetric cryptography algorithm whose performance is higher than the ECC. It aggregates data from the CHs and is then sent to the following entities:

#### 4.5.1. Classifier and Queue Manager

**Figure 3:**
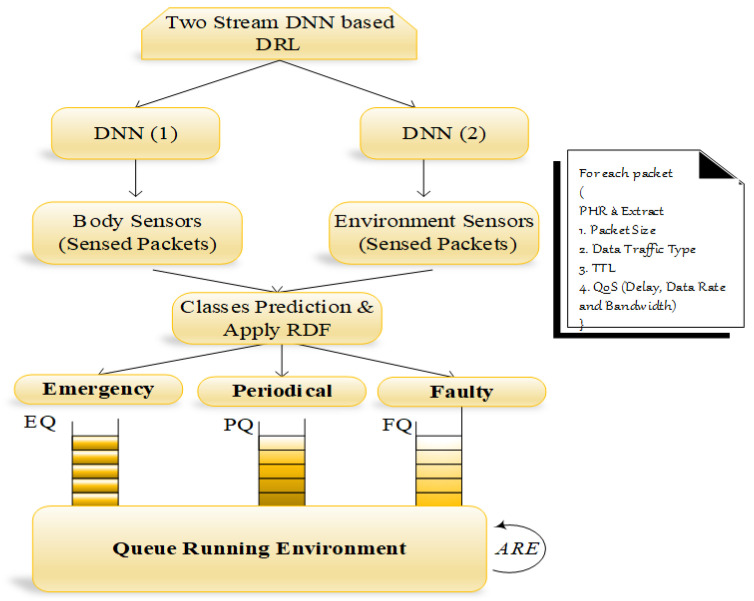
TS-DNN environment.

In this entity, aggregated data are classified into three classes, namely, emergency, periodical, and faulty data, using the two stream deep neural network (TS-DNN) in deep reinforcement learning. TS-DNN determines the classes from the multiple available inputs: packet size $P_{s}$, data traffic type $DT_{t}$, time to live TTL, health parameter, and *QoS* constraint (delay, data rate and bandwidth) $QoS_{CS}$. In the first stream of DNN, body sensor information is forwarded, whereas in the second stream of DNN, environmental sensors are forwarded. In this architecture, BAN sensed and environmental information is considered for classification. Each DNN consists of three kinds of layers: an input layer, hidden layers and an output layer. The flow and working of the classifier and queue manager are depicted in Figure 3. The process of each layer in DNN (1) and DNN (2) is illustrated in the following. The TS-DNN is explained in Algorithm 1.

- **Input Layer**: This is the first layer that is processed with the input neurons, and BAN packets and environment events are forwarded to the input layer of DNN (1) and DNN (2), respectively. Thus, the sensed packets from the body and environment sensors are represented as follows:
  (6) $$b\left(s\left(i\right)\right)=\left\{b{\left(s\right)}_{1},b{\left(s\right)}_{2},\cdots ,b{\left(s\right)}_{n}\right\}$$
  (7) $$e\left(s\left(i\right)\right)=\left\{e{\left(s\right)}_{1},e{\left(s\right)}_{2},\cdots ,e{\left(s\right)}_{n}\right\}$$
- **Hidden Layers**: Different numbers of computing weight values for each input parameter of the sensed packets. Based on the input requirements and the expected result, the number of hidden layers is defined. In the previously mentioned parameters, the fitness value is computed in hidden layers. Finally, the fitness function F(f) is expressed as follows,
  (8) $$F\left(f\right)=\left(\sum_{S=1}^{n}\frac{\tau_{x}+\mu_{x}+\alpha_{x}}{\Gamma_{x}\left(C_{1},C_{2},C_{3}\right)}\right)$$
  where *S* refers to the sensor nodes of n numbers (i.e., $\left\{S=1,2,\ldots ,n\right\})$, $\tau_{x}$ is the packet size weight value, $\mu_{x}$ is the different traffic type weight value, $\alpha_{x}$ is the *TTL* weight value, $\Gamma_{x}$ is the weight value for *QoS* constraints and C1,C2,C3 represents the delay, data rate and bandwidth.
- **Output Layer**: This layer predicts the classes according to the aforementioned parameters. It chooses the optimal class from the fitness values computed for each packet. Mathematically, it is formulated as follows,
  (9) $$O\left(L\right)=\vartheta \left(F{\left(f\right)}_{m}\vartheta \left(F{\left(f\right)}_{M-1}\vartheta \left(\cdots \vartheta \left(F{\left(f\right)}_{1}\right)\right)\right)\right)$$
  where ϑ is the activation function.

**Algorithm 1** TS-DNN.
1:
**Input:** $b\left(s\left(i\right)\right)=\left\{b{\left(s\right)}_{1},b{\left(s\right)}_{2},\cdots ,b{\left(s\right)}_{n}\right\}$

2:

$e\left(s\left(i\right)\right)=\left\{e{\left(s\right)}_{1},e{\left(s\right)}_{2},\cdots ,e{\left(s\right)}_{n}\right\}$

3:**Output:** Three Classes4:
**Begin**
5://TS-DNN algorithm using DRL6:Initialize all *b(s(i))*, and *e(s(i))* for Q(S,a)7:Repeat (For Each Episode)8:Choose *a* from *S* by policy derived from Q9:
**Input Layer**
10:
**for** $\forall BAN_{i}$ **do**
11:   Take action *a* and observe *r* and *s*12:   $Q\left(S,a\right)\to Q\left(S,a\right)+\tau [r+\gamma Max_{a},Q\left(S^{{}^{\prime }},a^{{}^{\prime }}\right)-Q\left(s,a\right)]$13:
**end for**
14:
**for** $\forall S_{i}\in S,$ **do**
15:   Learn ←τ,Γ,μ,ζ16:   Compute $F(f_{i})$17:   Assign $F(f_{i})\to$weightfunction18:
**end for**
19:Collect $F(f_{i})$ from all neurons20:Compute *O*21:Return (classes)22:
**End**



We classify the fault data by considering training set values. For the currently transmitted packet, we compute the relative difference factor (*RDF*), which shows the incorrect sensor readings. The notation RDF is calculated by,

(10) $$S\left(sr\left(c\right),\left(p\right)\right)=\frac{Sr(c*p)}{\left|c\right|\times \left|p\right|}=\sum_{i=1}^{n}\frac{i(c*p)}{\sqrt{\sum_{i=1}^{n}C_{i}^{2}}*\sqrt{\sum_{i=1}^{n}P_{i}^{2}}}$$

where Sr(c∗p) denotes the sensor readings for current and previous packets. After the classification, each class of the packets is put into a separate queue, and it is organized by the queue manager so that we nearly used three queues: emergency queue (EQ), periodical queue (PQ), and faulty queue (FQ). The FQ packets are used to find the *RDF* and send the alarm message to the particular sensor via PBA. To process all medical packets without any packet drop, we proposed Reyni entropy Rϵ, which runs all packets and changes the processing of one queue into another queue by computing the input parameters. Rϵ is the order of δ, where δ≥ 0 and δ≠ 1 are defined as,

(11) $$H_{\alpha }\left(x\right)=\frac{1}{1-\alpha }log\left(\sum_{i=1}^{N}P_{i}^{\alpha }\right)$$

where *x* is the discrete random variable with the possible result as 1,2⋯n and corresponding probabilities are $p_{i}=\frac{1}{n}$ for all *i*...*n*. Then, all the Renyi entropies of the distribution are equal: $H_{\alpha }\left(x\right)=logn$, and $P_{i}^{\alpha }$ represents the input parameters current values and α is the weight value, which ranges between 0 and 1. Finally, the packets are transmitted to the physician, ambulance, caregiver and pharmacy to perform further actions.

#### 4.5.2. Security Manager

In this entity, classified packets are encrypted using the extended-PRESENT algorithm, which is a lightweight blockcipher encryption algorithm. This extended version uses fast bit permutation instruction, which consumes less energy than the traditional PRESENT and AES algorithm. This algorithm consumes a small amount of memory in all programmed devices due to its reduced size which also minimizes the computation time when compared with other cryptography algorithms.

**Figure 4:**
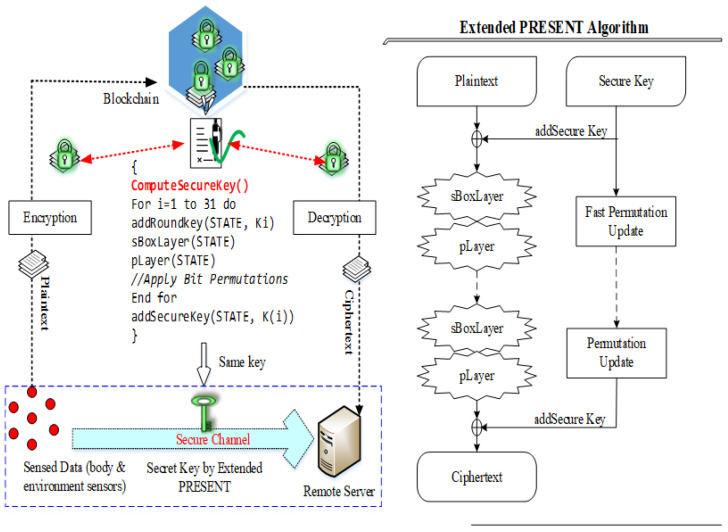
Extended version of PRESENT algorithm.

To mitigate the issues of PRESENT, i.e., low security strength (comparatively higher than previous symmetric algorithms), we proposed a modified algorithm in which permutation operation is changed with respect to the fast bits based permutation operation. This step further reduces energy consumption and provides higher security and reliability to the body sensors. In the first step, a secret key is calculated using patient input parameters such as *ID*, *PWD*, location and biometric records. However, key generation is implemented using two types of keys such as 80 bits and 128 bits. Each type of key strength is used in 31 rounds of operation which performs the XOR operation that introduces the round key $K_{i}$ for 1≤i≤32, where $K_{32}$ is used for post whitening. In this paper, 80 bits of key size are used and the proposed PRESENT algorithm is presented in Figure 4. Computing a security key for the cluster is innovative for this work in which the PRESENT algorithm uses the dynamic keys of cluster members for key generation.

Let us assume that a cluster with *M* members is $S_{1},S_{2},S_{3},\cdots ,S_{M}$. Every sensor node consists of a secret key as $SK_{1},SK_{2},SK_{3},\cdots ,SK_{M}$ consistently. In the proposed work, the CH is intended for generating ot−ℵ in each round R. For that, it put on XOR operations to the secret keys for its member nodes. ot−ℵ is generated at time $t_{1}$ as follows,

(12) $$ot-ℵ=SK_{1}⊕SK_{2}⊕SK_{3}\cdots ⊕SK_{M}$$

Similarly, if the cluster is organized with dynamism due to less energy and link quality, then the CM’s table is adapted according to the CH. Consequently, in the next round, the CH generates ot−ℵ with new CM keys. In this way, the cluster key is changed at each time. The generation of ot−ℵ achieves encryption as in the PRESENT algorithm. The $SK_{i}$ is XOR with the packets on every round that is caused by the previous round. First, the plain text is XOR with t−ℵ, and then ot−ℵ is shifted and provides the security key. The first part of the XOR text is again over XOR with the round key. In this way, 31 rounds are applied to generate the ciphertext for aggregated packets. The pseudocode for the PRESENT algorithm is described in Algorithm 2.
**Algorithm 2** OT-PRESENT algorithm.1:**Input:**$Packets\;P_{\left(i\right)}\;and\;SK_{1},SK_{2},SK_{3},\cdots ,SK_{M}$2:**Output:**$Ciphertext\;of\;P_{\left(i\right)}$3:**Begin**4:// OneTime Security Key generation5:**for**$\forall S_{i}\in S$**do**6:   $Prepare\leftarrow SK_{1},SK_{2},SK_{3},\cdots ,SK_{M}$7:   Create←ot−ℵ8:**end for**9:// PRESENT encryption10:R← 1 // R= round11:state← Plaintext (P)12:**While** (R < 31),13:SK=ot−ℵ14:state←SK⊕State15:state←SBoxLayer(State)16:// Extended-PRESENT uses revised Permutation Function17:state←FastBitpermutationlayer(State)18:R←R+1, do19:R←rightCircularShift(ot−ψ,19)20:SK[76−79]←SBoxLayer(Key[76−79])21:SK[15−19]←ot−ℵ[16−19]22:**end do**23:**End While**24:**if** (R=31), **then**25:   lastSK←generateKey(Key,R)26:   State←State⊕lastSK27:   $Ciphertext\;of\;P_{\left(i\right)}\leftarrow State$28:**end if**29:**End**

The pseudocode of the proposed OT-PRESENT algorithm is depicted for 80 bits of key strength. The proposed extended version of the PRESENT algorithm uses quite simple XOR operations that are comparatively lightweight than traditional PRESENT and other symmetric algorithms. Furthermore, this algorithm increases the security level of the system.

#### 4.5.3. Channel Selector

Upon receiving the critical data, an idle channel is selected to transmit the data without delay, whereas periodical data are forwarded via the best channel. It is determined by the multi-objective-based channel selection scheme from the multiple inputs: RSS, $Re_{ss}$, SNR $s/n_{r}$, channel capacity $C_{ct}$ and radio power $R_{pr}$.

(13) $$RSSI=t_{p}+a_{g}-p_{l}$$

*RSSI* is a significant parameter that measures the total received signal with respect to the noise. *RSSI* is computed using $t_{p}$ and $a_{g}$ which are transmit power and antenna gain, respectively, and $p_{l}$ is the path loss.

The best channel is chosen for forwarding the periodic packets. $Re_{ss}$ represents the sum of the average of the total power obtained from the specific antenna. The higher the signal value is, the higher the RSS. The SNR estimates the value of accurate signals based on the noise present in them.

The signal-to-noise ratio measures the ratio of the valid signal with respect to the noise present in it. The parameter $s/n_{r}$ is computed as follows,

(14) $$\frac{s}{n_{r}}=10{log}_{10}\frac{P_{sg}}{P_{Ns}}$$

The terms $P_{sg}$ and $P_{Ns}$ denote the amount of signal and noise present in a particular signal. Higher values of $s/n_{r}$ imply the presence of more signal than noise. Furthermore, the channel capacity of the signal is computed mathematically using Shannon’s theorem given below,

(15) $$C_{ct}=b_{w}*{log}_{2}\left(1+s/n_{r}\right)$$

Where $b_{w}$ is the bandwidth, which is identified as the amount of data transfer rate that exists in the channel. On estimates, the three significant parameters of a channel and the best channel are selected. The radio power consumption is different for each packet, and hence, the dynamic value of the radio power is computed in the running state. The determined channel ensures successful data transmission without any delay or loss. With the use of channels, packets are encrypted at the PBA and forwarded to the corresponding sink node.

Finally, the data reach the blockchain according to the data traffic type. If the sensed packet is critical, it is first sent to the healthcare unit and then stored into a cloud server, whereas the periodical data are stored in the cloud server and sent to the healthcare unit.

## 5. Experimental Results and Discussion

In this section, we illustrate the performance of the proposed B-DEAH architecture. This section is categorized into three sections: simulation environment, comparative analysis and security and efficiency analysis of the proposed B-DEAH architecture based on the existing works.

### 5.1. Simulation Environment

The proposed B-DEAH model is constructed using the OMNeT++ simulator, which is suitable for body area networks. The programming language supported in this tool is C++. Furthermore, WBAN is modeled by the network description (NED) language, and it uses the special network module MiXiM. It provides useful built-in modules such as battery, channels, packets, messages, mobility, mac, PHY, network models, application layers and several examples for WBAN. The role of the physical WBAN environment is to establish the data transmission from the source to the destination. In this work, five WBANs are considered, and each WBAN has distinct characteristics as follows,

- Communication Type: Off-body, on-body, body to body, off to off;
- Scenario Condition: Same room.

**Figure 5:**
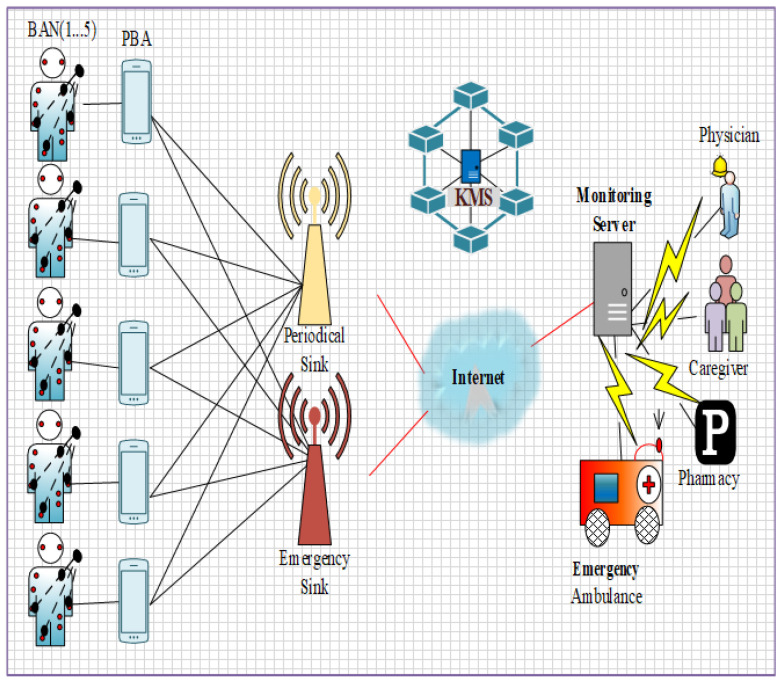
Network topology.

The simulation environment is illustrated in Figure 5. The following table represents the simulation configuration for the proposed B-DEAH model. Furthermore, a detailed description of body sensors for a single BAN configuration is represented in Table 4.

**Figure 6:**
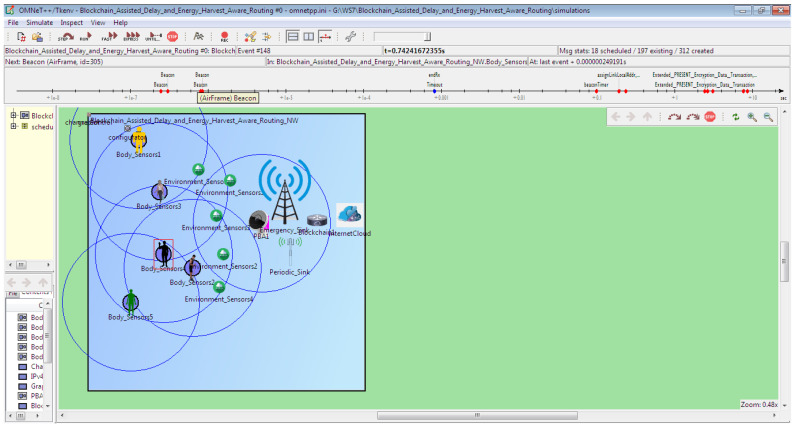
OMNeT++ simulation environment for multiple ban and body sensors in single ban.

Figure 5 shows the simulation setup for the proposed B-DEAH using the OMNeT++ simulator. Figure 6 shows the network construction, and their components are represented. Likewise, each BAN user is connected with the two sink nodes for both emergency and periodical data transmission via PBA. Based on the sensed data transmitted to the sink nodes, the analysis was taken into account for the proposed work. The performance analysis, such as *QoS*, security and energy efficiency, is described in the following section in detail. The considered body sensors used for the implementation of B-DEAH are adjusted with the dynamic threshold values for data type differentiation. Therefore, emergency and periodical values for each body sensor are computed and depicted in Table 5.

**Use case (Cardiac Vascular Disease):** Body sensors are specifically developed for the purpose of collecting, monitoring, diagnosing and controlling human medical information. From the body sensors, various health symptoms are periodically collected or in an emergency state. A heart attack is a serious threat in almost all countries. Among all the types of heart attacks, cardiovascular disease is the most prevalent disease worldwide. For this disease, the death rate is growing gradually every year. In this paper, the application of *cardiac vascular disease* is demonstrated for patients.

**Figure 7:**
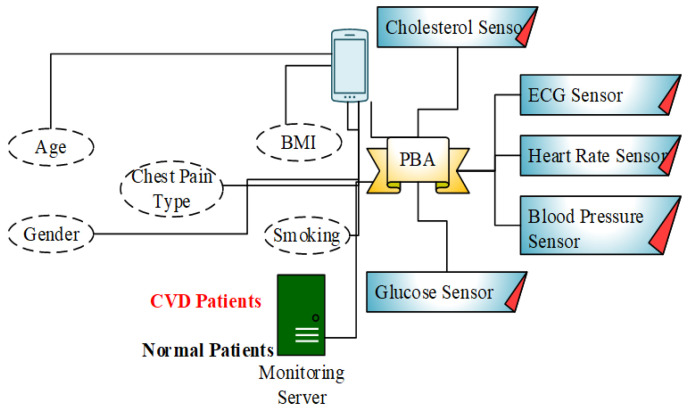
Block diagram for CVD patients diagnosis.

The prototype testing uses distinct sensors such as Heart BPM, ECG, blood pressure, glucose, cholesterol, etc. The B-DEAH architecture is designed with node clustering, data aggregation, and secure data transmission. In the PBA, emergency events are predicted and transmitted to the cloud server and corresponding authority (ambulance, doctor or clinics) via the emergency sink node. Sequential communication is performed, which is started at the body sensors. An overview of the prototype model is represented in Figure 7. After the collection of all sensed data from sensors, it is forwarded to the PBA through Bluetooth or Wi-Fi. The PBA also collects the rest of the patient’s parameters, such as age, sex, chest pain type, BMI, and smoking history. Finally, all the physiological parameters are transmitted, classified and used to predict the cardiac severity status of the patient and sensed data types as emergency and periodical results to the patient.

### 5.2. Comparative Analysis

In this section, the details of the simulation results are discussed in detail. The proposed B-DEAH is compared with three previous works, CF-EHARP, PCA and E-HARP. The main sets of parameters that are taken into account for comparison are network throughput, end-to-end delay, packet loss rate, authentication time, residual energy and success rate. Furthermore, the efficiency of the work is compared with the previous works with respect to fault data elimination check (%) and reliability (%) to the previous works.

#### 5.2.1. Network Throughput

**Figure 8:**
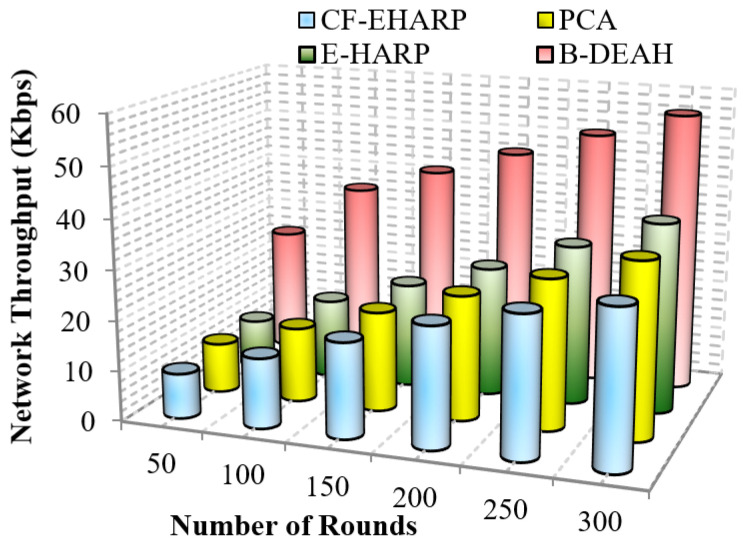
Network throughput vs. simulation rounds (emergency packets).

**Figure 9:**
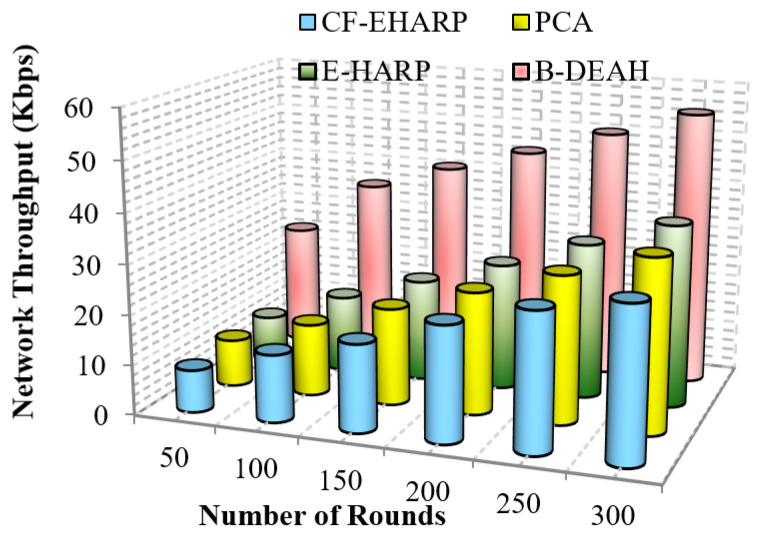
Network throughput vs. simulation rounds (periodic packets).

It is defined as the sum of data transmitted successfully to the destination node. However, the increase in throughput shows higher performance. It is an important *QoS* metric that shows the quality of the network in data transmission. Hence, maximizing the *QoS* performance is important in WBAN. Mathematically, it is calculated as follows,

(16) NetworkThroughput=P(s)/T(t)

where P(s) is the size of the arrived packet and T(t) is the transmission time for a single packet. Figure 8 and Figure 9 represent the throughput analysis for emergency and non-emergency packets, respectively.

From the results obtained, it is proven that when the number of rounds increases, the network throughput increases gradually for the proposed B-DEAH work. However, the throughput performance is affected by long route acquisition delays, high routing overhead, high traffic over a particular route, higher rated buffers, and so on. In PCA, a large number of nodes are deployed but less effective in data transmission. The larger communication overhead introduces low throughput. In the E-HARP protocol, nodes are sparsely distributed, and communication is simple, which results in higher throughput than PCA and B-DEAH. In the proposed B-DEAH method, efficient routing is performed by conducting clustering using the SHO algorithm, which increases the throughput, thereby increasing the *QoS*.

The extracted routing information for communication leads to higher throughput in the B-DEAH model. For example, PCA attained 22.5 Kbps for 300 rounds, which is 50% less than B-DEAH, and other works have more than 50% lower performance than B-DEAH. Due to the path selection by the MOORA algorithm, throughput is obtained in a higher range. For both, emergency and periodical packets are running simultaneously since two different kinds of sink nodes are deployed for individual packet transmission. In B-DEAH, *RDF* is utilized, whose main role is to remove the fault data packets over a path and thus eliminate the transmission of the faulty packets. Therefore, throughput is increased when the network rounds also increase.

#### 5.2.2. End-To-End Delay

It is a time-based *QoS* metric that computes packet transmission between the source and the end server. It computes in all stages after the packet is sensed at the source level. Three kinds of delays are computed: propagation delay, processing delay, transmission delay and queuing delay. The mathematical formulation for the delay is computed as follows,

(17) $$End-to-End\;Delay=\sum_{P_{i}\in P}T_{R(P)}-\sum_{P_{j}\in P}T_{S(P)}$$

where $T_{R(P)}$ is the sum of time taken for packets reception and $T_{S(P)}$ is the sum of time taken for packets transmission.

**Figure 10:**
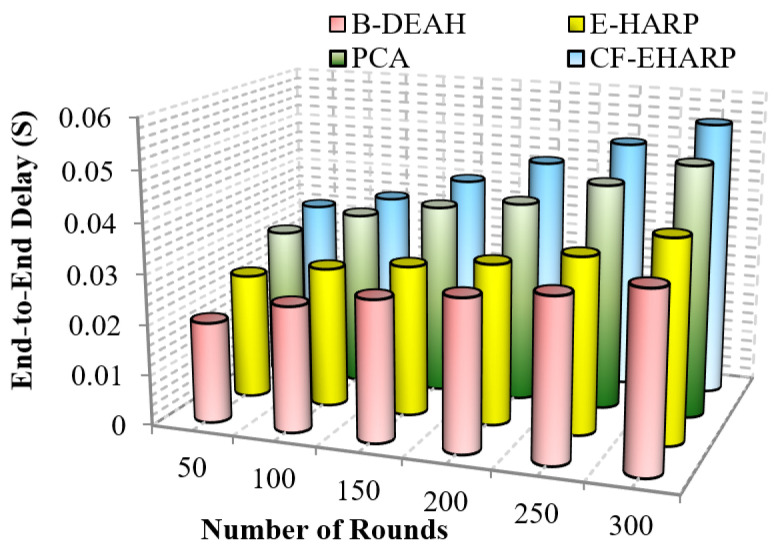
End-to-end delay vs. simulation rounds (emergency packets).

**Figure 11:**
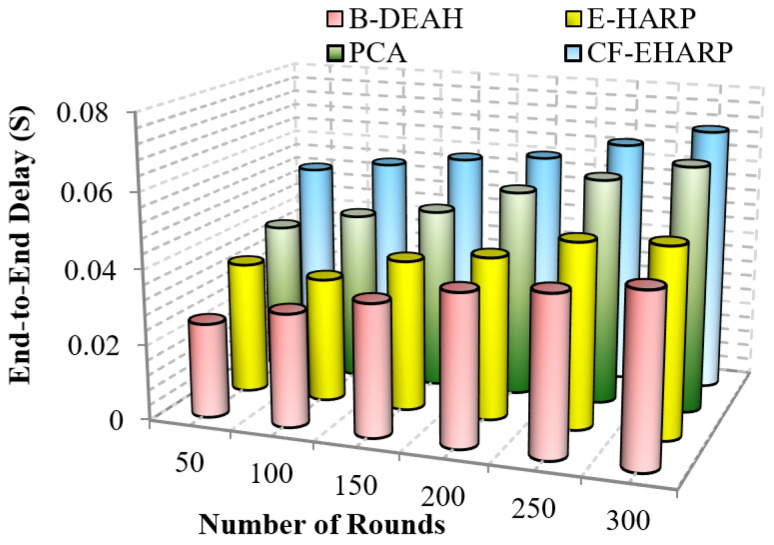
End-to-end delay vs. simulation rounds (periodic packets.

Figure 10 represents the efficiency of delay for emergency packets. It is analyzed for the number of simulation rounds. Delay increases gradually according to the simulation rounds since the number of rounds depends upon the number of communications to the destination. In Figure 11, the delay in B-DEAH is much smaller than that in previous works. A conflict between emergency and nonemergency packet transmission is avoided in this work since emergency packets are transmitted to the emergency sink and then to the monitoring server. Hence, the waiting time of each packet is reduced by 50% compared to previous works. Furthermore, emergency data are transmitted to separate channels and nonemergency, i.e., periodical data packets forwarded using the best set of predicted channels. Thus, the delay in channel selection is minimized in the proposed B-DEAH method.

Important (critical) messages must be transmitted more quickly than nonimportant messages. In previous works, delay increased due to the interference between channels. The delay difference between previous works is 15% less than PCA, 27% less than E-HARP and 50% less than CF-EHARP. The delay in the E-HARP is reduced due to the error correction mechanisms. After error correction, original packets are transmitted between the source and the destination. Similarly, PCA does not invoke any attackers in between the route so that the overhead induced by the attackers is reduced. In the following, the performance delay is computed for periodic packets.

#### 5.2.3. Packet Loss Rate

However, the packet loss rate must be lower to show that the network has obtained higher *QoS*. In general, the packet loss rate is reduced due to the lack of awareness of the buffer rate and network size. Furthermore, the rate of packet drop increases when malicious node presence is high, and in this case, wireless channels are vulnerable. Lack of knowledge about the network increases the packet loss rate. This metric is computed by the total number of packets transmitted from the source to the number of packets sent by the destination.

**Figure 12:**
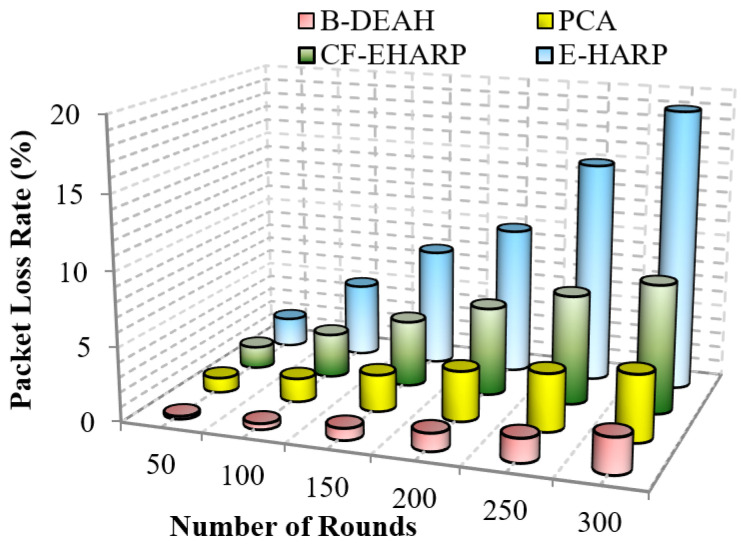
Packet loss rate vs. simulation rounds.

A comparison of the packet loss rate with respect to the simulation rounds is depicted in Figure 12. The proposed B-DEAH architecture addresses all the previous issues of higher packet losses. An optimal channel selection method is introduced by considering multiple objectives, such as RSS, SNR, channel capacity and radio power. All sensed packets from body sensors are represented with their *QoS*, and reliable transmission of packets occurs between the source and destination pairs. Previous works E-HARP and CF-EHARP are loss packets without the information of packet type. In B-DEAH, two sinks are deployed in which PBA forward emergency requests to the first sink and nonemergency requests forward to the second sink, which reduces the packet loss rate efficiently.

#### 5.2.4. Authentication Time

**Figure 13:**
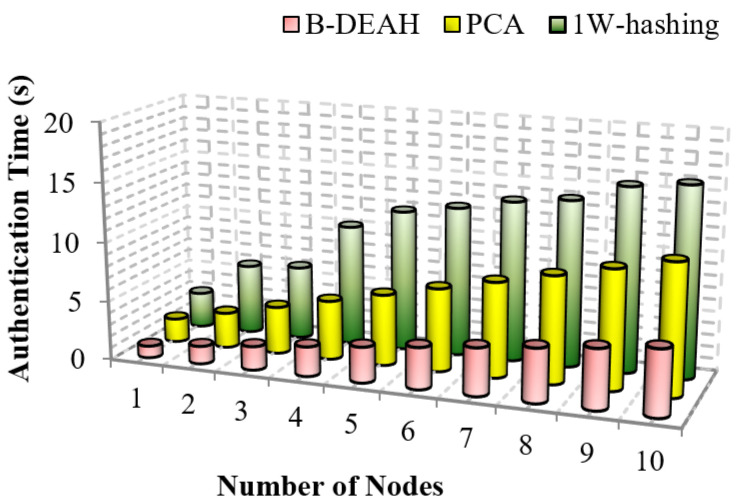
Authentication time vs. number of nodes.

It is an attack mitigation metric that calculates to avoid intrusions from failures as authentication time. The authentication procedure is different for symmetric and asymmetric algorithms. When compared to the asymmetric, symmetric algorithms consume less energy since they are fast at default. The comparison of authentication time for B-DEAH and 1W-hashing is depicted in Figure 13.

From Figure 13, we can prove that the proposed B-DEAH scheme requires less time for authentication. In PCA, traditional algorithms are used for encryption, i.e., ECC, which consumes more time and energy from sensors. Authentication time must be less for any kind of security mechanism. In 1W-hashing, smartcard-based authentication is implemented by a hashing algorithm. Our proposed three factored security scheme has had a greater impact than previous works. Key registration and authentication are efficiently performed in the proposed work with lightweight algorithms, which reduce the authentication time and increase security. This comparison confirms that the proposed B-DEAH architecture ≅65% reduces the authentication time.

#### 5.2.5. Residual Energy

It is a major parameter in WBAN that measures the energy efficiency of the system. The initial distribution of energy is equal for all sensors. The main issue in perceiving WBAN communication is to manage the residual energy of body sensors. The residual energy can be varied in accordance with the time, packet size and number of rounds of processing. Furthermore, based on the connectivity to the neighbor sensors and coverage, residual energy values are different. The shorter residual energy consumption for each processing, such as sensing, transmission and reception, proves that the model is energy efficient. A mathematical formulation of residual energy is given below,

(18) $$R_{E}\left(i\right)=\frac{\left(E_{node}\times E_{init}\right)}{100}$$

where $E_{node}$ is the residual energy consumption rate of the node, $E_{init}$ represents the energy level at the initial stage. However, the transmission energy is directly proportional to the distance from the source to the destination.

**Figure 14:**
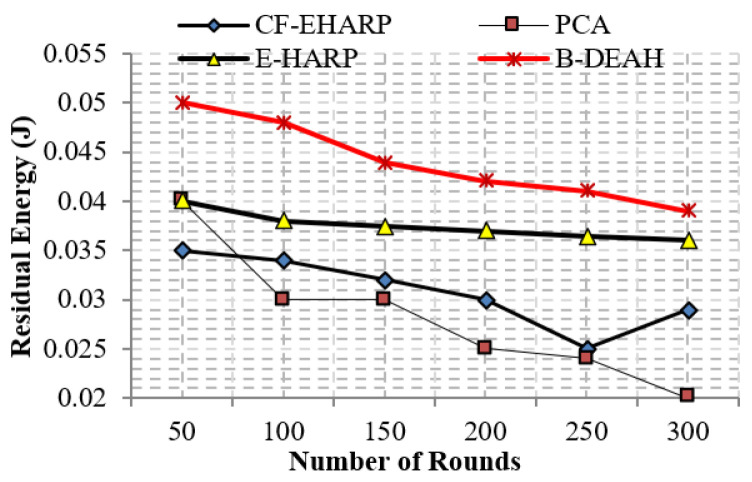
Residual energy vs. simulation rounds (emergency packets).

**Figure 15:**
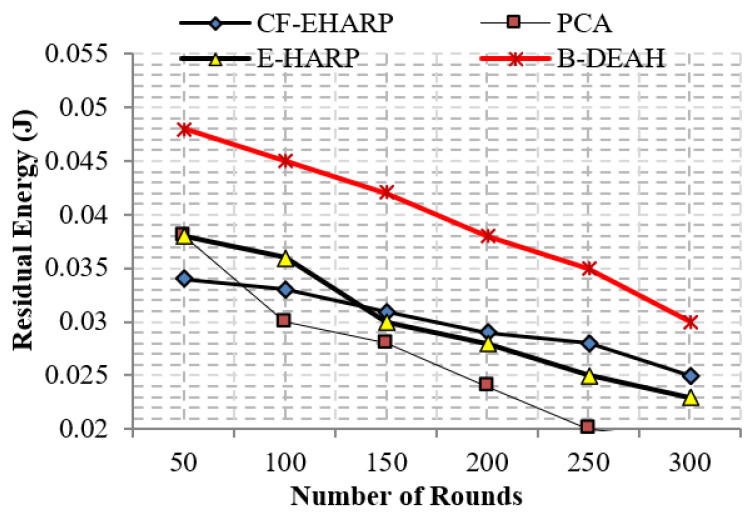
Residual energy vs. simulation rounds (periodic packets).

**Figure 16:**
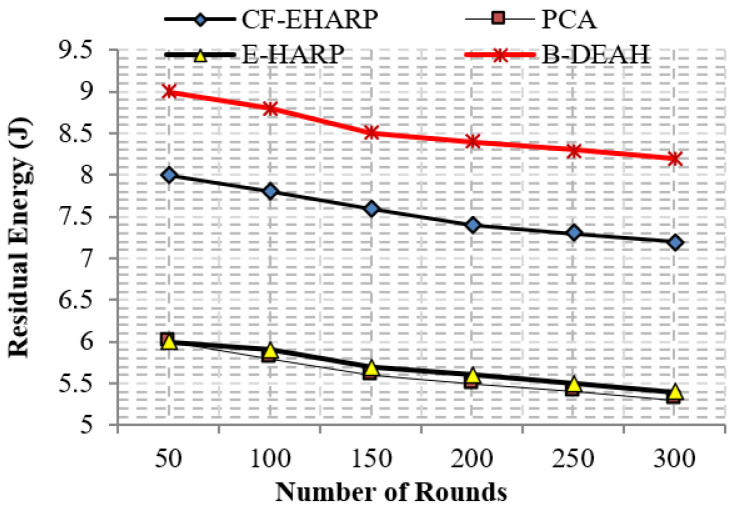
Residual energy vs. simulation time (s) (emergency packets).

**Figure 17:**
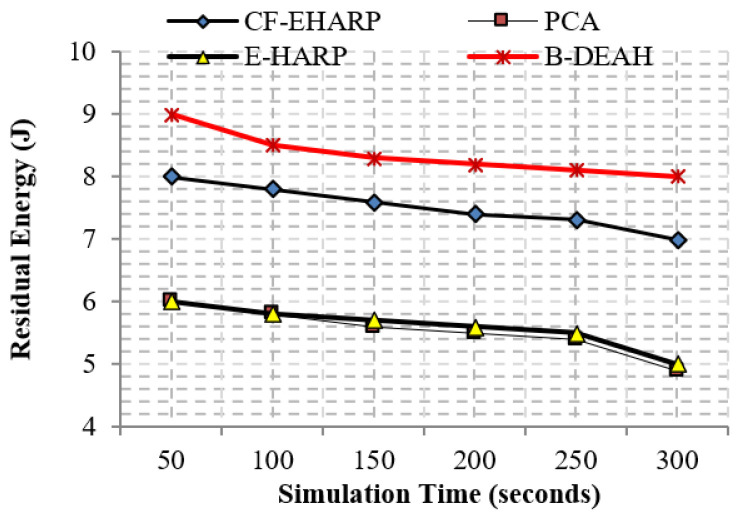
Residual energy vs. simulation time (s) (periodic packets).

The residual energy defines the remaining energy of a sensor after transmitting a single packet. As per the increase in the number of rounds, residual energy gradually decreases. Residual energy is compared with the two parameters, simulation rounds and simulation time, for both emergency and nonemergency packets. Previous works are reduced to the lower level of residual energy at 50 rounds. Designing a lightweight framework increases the *QoS*, which is achieved by three kinds of mechanisms: security by blockchain technology, fault data elimination by *RDF*, and two sink deployment for emergency and nonemergency packet transmission in the proposed B-DEAH method, which increases the residual energy, thereby increasing the energy efficiency. Figure 14 and Figure 15 represent the residual energy in terms of simulation rounds for emergency and periodic packets. Figure 16 and Figure 17 represent the residual energy in terms of simulation times for emergency and periodic packets.

The proposed B-DEAH is 40% better than CF-EHARP, PCA and E-HARP. For every communication, the residual energy of each sensor and relay sensor is considered for multihop-based routing. The higher residual energy gives the higher network lifetime, and the average behavior of the proposed work confirms that it does not take higher residual energy and gives the stochastic nature of higher residual energy availability.

#### 5.2.6. Success Rate

This parameter represents the success rate of data transmission over time. Due to collision and malicious node involvement, packet transmission is changed and fewer packets are successfully reached to the destination. In this work, we compute the performance of the success rate for the proposed B-DEAH and the existing works. Due to BAN mobility, the success rate is often changed. fall into a lower level. To address this issue, proper network design is an important part of any kind of network. A successful transmission shows no collision, no attackers and no interference in channels. Mathematically, the success rate is computed as follows.

(19) $$SucessRate=\frac{\#\;of\;sent\;packets}{\#\;of\;received\;packets}$$

**Figure 18:**
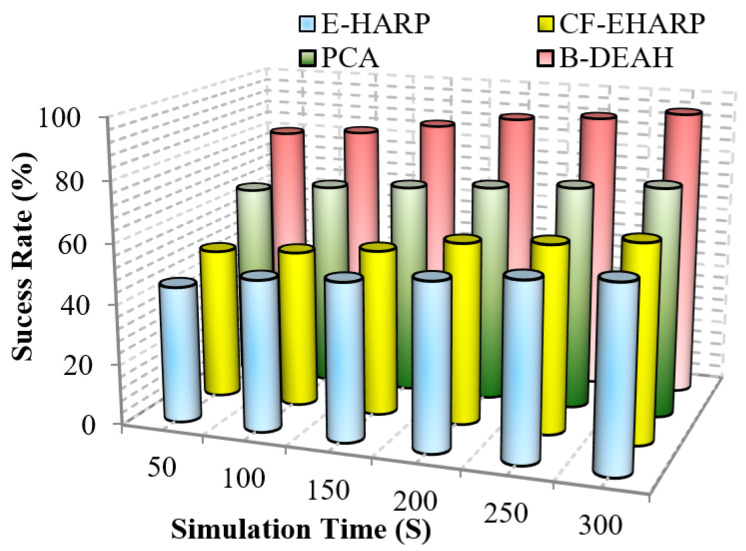
Success rate vs. simulation time (emergency packets).

**Figure 19:**
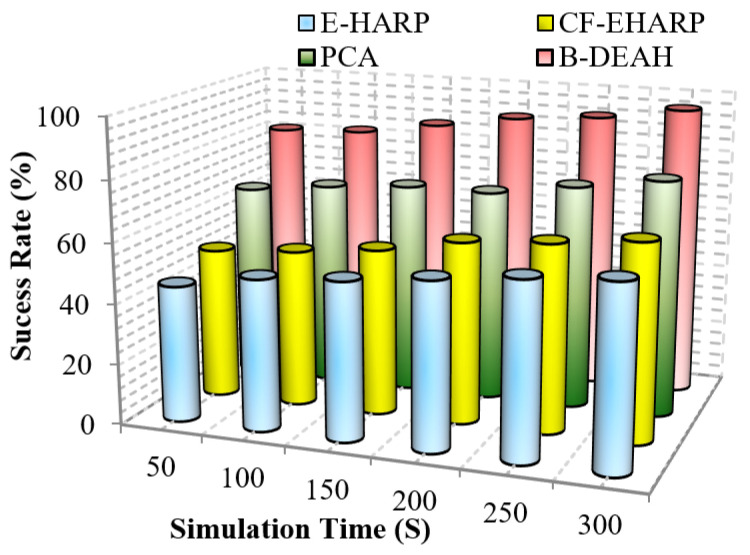
Success rate vs. simulation time (periodic packets).

Therefore, the packet transmission success rate is computed using the total number of packets sent and the total number of packets received successfully. Figure 18 and Figure 19 represent the success rate of the proposed B-DEAH model with respect to the simulation time for the previous works.

As per the route selected, packets are transmitted to the CH, and packet typewise communication is established to the sink nodes via PBA. In the emergency sink, packets are forwarded without any waiting delay. In previous works, a single sink-based packet transmission decreased the success rate. In blockchain-based security and *QoS* provisioning addresses, all the research challenges, such as packet tampering and authorization via weak credentials, are eliminated. From Figure 18 and Figure 19, it is perceived that the proposed B-DEAH succeeded with a higher success rate, and previous works obtained a 40% lower success rate.

### 5.3. Security & Efficiency Analysis

In this section, the security and efficiency of the work are discussed for the proposed as well as previous works.

By increasing the strength of security, the proposed work is more secure against different attackers. Apart from the CVD, the proposed model is capable of ensuring high *QoS*, security and energy efficiency for many disease diagnosis applications. In the following, we discuss the attacks mitigated in this system.

- *BAN Impersonation Attack:* In this attack, unauthorized patients attempt to submit and access data from the cloud. However, the sensors from the body and environment are compromised by the attackers. The authentication to the blockchain cannot be compromised by an attacker and cannot be passed until the correct credentials are submitted.
- *Flooding Attack*: In this attack, more illegitimate requests are forwarded by attackers. Hence, sink nodes and any other communication devices cannot tolerate a high number of requests. For each BAN, the PBA’s role is to monitor and audit the abnormal packets and notify this information to the blockchain and remote server.
- *Path-based DoS Attack*: DoS is a denial of service attack in which attackers’ behavior is to exhaust the resources for sink nodes and other devices and creates a busy route. This makes the huge delay in processing legitimate packets, and hence, the emergency packet delay is higher and leads to more packet losses.

The efficiency of the work is analyzed using two parameters: fault data elimination check and reliability. The comparison analysis of these two metrics is represented in the following.

#### 5.3.1. Fault Data Elimination Check

The proposed B-DEAH incorporates a fault data elimination check because the IoT-based WBAN environment produces fault measurements, which are very high; therefore, it is avoided using *RDF*. Earlier works concentrated on packet transmission and energy consumption. The B-DEAH architecture determines the similarity between two or more histories of old packets in the database. B-DEAH considers all packet header information. The performance of the fault data elimination check is evaluated for B-DEAH and E-HARP. From the analysis, it is confirmed that fault data packets are removed. Table 6 shows that B-DEAH attains the performance of the fault data elimination check. Table 7 shows the overall comparison of proposed and existing works.

However, some intended packets are not determining the fault data in earlier works. This mitigation aids in effective data aggregation and classification for minimizing the end-to-end delay and energy consumption.

#### 5.3.2. Reliability

**Figure 20:**
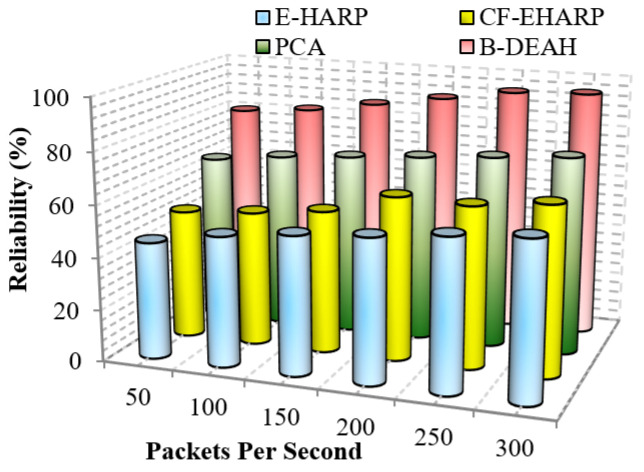
Reliability vs. packets per second.

This metric evaluates the reliability provided by the presented schemes. This metric is determined as the total change rate of the packets sent to the destination. This metric is defined as the ratio between the number of packets affected or modified by the attackers and the total number of packets transferred in the destination. This metric is high when the overall system is free from attackers and congestion overhead. Figure 20 depicts the performance of the reliability for the proposed B-DEAH and previous works such as E-HARP, CF-EHARP and PCA. As a result of lightweight security schemes based on necessary input criteria for all processes, the reliability of the proposed work is achieved.

## 6. Conclusions and Future Work

This paper proposes a new patient remote healthcare monitoring system for addressing the existing issues. The proposed B-DEAH model is designed for three types of communications such as intra-WBAN, inter-WBAN and beyond-WBAN. For decentralized communication and security, blockchain is deployed in an IoT-based WBAN environment. A three factored security algorithm is proposed for patients registration, security credentials are forwarded to *KMS*. For data transmission and energy consumption reduction, the clustering process is initiated. Spotted hyena optimizer is used for clustering that computes the best CH using the fitness of prey and updates position accordingly. Then intracluster routing is implemented using the MOORA algorithm. It improves the performance of packet success rate and avoids packet losses and delays. In PBA, three operations are used such as classification and queue management, security provisioning and channel selection by TS-DNN in DRL, four-Q-curve for PBA authentication and packets encryption using the PRESENT algorithm, and multi-objective based channel selection algorithm. Then packets are forwarded to the emergency and periodical sinks in accordance with the priority. The simulation is conducted for various performance metrics as *QoS*, energy efficiency and security. Based on the results noted, the proposed B-DEAH provides better performance than previous works. In the future, we intended to focus on the following aspects,

- Investigation of mobility since mobility is a crucial parameter of BAN. The human body parts are in motion constantly. Here, we planned to use handover mechanisms for mobility management.
- Duty cycling MAC scheduling is studied for managing the energy level of each body sensor.
- Furthermore, other emerging medical diagnosis applications are concentrated for user physiological parameter analysis, such as diabetes, asthma, Parkinson’s disease, or COVID-19.

## Figures and Tables

**Table 1:** Research gaps.

Area Focused	Research Gap
*QoS* Achievement [28,29,30,31,32,33,34]	Direct data transmissions without considering clustering to the sink nodes were achieved in some of the existing papers which affect the *QoS* in terms of high latency. Many of the existing papers perform clustering however, the clustered data in the gateway was not efficiently handled in terms of delay and throughput which also affects the *QoS*. The routing protocols used in the existing research are limited with less reliability in communication and suffer from transmission errors which also affect the *QoS*.
Security Provisioning [36,37,38,39]	Some of the existing works not considered authentication rather they consider data security which also affects the patient’s privacy. Most state-of-the-art works employ heavy-weight cryptographic algorithms for data encryption and authentication, however, which leads to high energy consumption in the WBAN-IoT networks. Some of the existing work limits with considering the minimal amount of security metrics for providing security in WBAN-IoT however, which imposes major attacks in the WBAN-IoT environment such as false injection attacks, impersonation attacks, etc.
Blockchain [41,42,43,44]	Conventional blockchain models are limited with high energy consumption, latency, and scalability issues in terms of block creation and validation time.These problems in the conventional blockchain are not suitable for resource-constrained WBAN-IoT.

**Table 2:** Patient healthcare data.

Data Collection Factors	IoT Devices	Parameters	Sensing Event Type
Body sensors related data	Heart sensors, Q-sensor, EEG devices, ECG monitor, smart wearable’s, Gastro sensors.	Heart Rate, Blood Pressure, Oxygen Saturation, Temperature, Blood Sugar, Respiration Rate, GI tract, ECG, EEG	High Heart Rate, High Blood Glucose Level, High Blood Glucose Level, Stress, Anxiety, and Restlessness
Environment related data	Temperature sensors, Humidity sensors, Chemical detectors, Noise sensors	Temperature, Air Quality, Noise Level, Toxic Waste	High room temperature, High noise level, High light intensity

**Table 3:** IEEE 802.15.6-based CSMA/CA-MAC Protocol Data Traffic Specification.

PHP (Octal Symbol)	${\mathrm{CWS}}_{\min}$	${\mathrm{CWS}}_{\max}$	Data Traffic Type
4	≅1	≅3	Emergency (VHP)
3	≅2	≅6	Periodic (HP)
2	≅4	≅8	Video (NP)
1	≅4	≅8	Voice (NP)
0	≅4	≅8	Others (NP)

Factors to adjust the ${CWS}_{min}$ and ${CWS}_{max}$ are distance, *RSSI*, residual energy. PHP—patient health priority, VHP—very high priority, HP—high priority, NP—normal priority.

**Table 4:** B-DEAH configurations.

Parameters	Specifications
**Simulation Parameters**	
Simulation Environment	1000 × 1000 m
Number of WBANs	1–5
Number of Body Sensors (Each BAN)	12
MAC type	IEEE 802.15.6 MAC
Sensing Interval	0.1 s
Multiple access technique	CSMA/CA
Packet size	512 bits
Bandwidth	20 MHz
Transmission rate	20 kpbs
Modulation (Data Rate)	DQPSK (1000 Kbps)
Energy consumption	0.5 mW
Simulation time	50 s
Number of Sink Nodes	2(1-Emergency, 2-Periodical)
Transmission Rate	5 Packets/s
Number of PBA (each WBAN)	5
MAC Header Length	32
Number of Frame Slots	20
Slot Duration	1 s
Buffer Capacity	32
Block Size	2 KB
Block Chain Type	Linear/Non-Linear
Key Size	80 bits
Passwords	Alphabets/integer
**System Setup**	
Operating System	Windows 7 (32-bit)
Processor	Dual core
RAM	4 GB and above

**Table 5:** Body sensors (emergency and periodical range).

Body Sensors	Emergency (Data Range Units)	Periodical (Data Range Units)
ECG	∼60–100 bpm	>100 bpm
Heart Rate	60–100 bpm	>100 bpm
Blood Pressure	120/80 mm/Hg	≥140 mm/Hg
Temperature	>100 °F	97.8–99 °F
Oxygen Level	<60 mm/Hg	80–100 mm/Hg
Respiratory	<6 bps	30–40 bps
EEG	<7 Hz	>8 Hz

**Table 6:** Fault data elimination check.

Number of BAN	Fault Data Elimination Check (%)	
	B-DEAH	E-HARP
1	97.6	80
2	98.6	81.5
3	99.3	82.5
4	99.6	83.6
5	99.8	85

**Table 7:** Overall comparison of proposed and existing works.

Performance Metrics			CF-EHARP	PCA	E-Harp	B-DEAH
Throughput (Kbps)		Emergency Packets	20.8 ± 0.4	22.5 ± 0.3	23.8 ± 0.5	42.5 ± 0.1
		Periodic Packets	20.08 ± 0.4	22 ± 0.2	23.11 ± 0.5	41.75 ± 0.1
End-to-end delay (s)		Emergency Packets	0.042 ± 0.3	0.039 ± 0.2	0.031 ± 0.4	0.028 ± 0.1
		Periodic Packets	0.06 ± 0.3	0.052 ± 0.2	0.041 ± 0.5	0.036 ± 0.1
Packet loss rate (%)		Number of rounds	5.18 ± 0.4	2.81 ± 0.2	9.83 ± 0.5	1.1 ± 0.1
Residual energy (J)	Simulation Rounds	Emergency Packets	0.03 ± 0.3	0.026 ± 0.2	0.03 ± 0.4	0.044 ± 0.1
		Periodic Packets	7.55 ± 0.4	5.6 ± 0.3	5.68 ± 0.5	0.039 ± 0.1
	Simulation time	Emergency Packets	7.51 ± 0.3	5.53 ± 0.2	5.6 ± 0.4	8.53 ± 0.1
		Periodic Packets	57.3 ± 0.4	70.83 ± 0.3	53.33 ± 0.5	8.35 ± 0.1
Success rate (%)		Emergency Packets	57.33 ± 0.3	70.83 ± 0.2	53.33 ± 0.4	87.5 ± 0.1
		Periodic Packets	57.33 ± 0.4	70.83 ± 0.2	53.33 ± 0.5	87.83 ± 0.1
Reliability (%)		Packets per second	57.83 ± 0.4	70.83 ± 0.3	53.5 ± 0.2	87.83 ± 0.1

## Data Availability

Not applicable.
